# The Harvey–Bradshaw Index Adapted to a Mobile Application Compared with In-Clinic Assessment: The MediCrohn Study

**DOI:** 10.1089/tmj.2018.0264

**Published:** 2020-01-07

**Authors:** Ana Echarri, Isabel Vera, Virginia Ollero, Claudia Arajol, Sabino Riestra, Pilar Robledo, Marta Calvo, Franscisco Gallego, Daniel Ceballos, Beatriz Castro, Mariam Aguas, Santiago García-López, Ignacio Marín-Jiménez, María Chaparro, Paco Mesonero, Iván Guerra, Jordi Guardiola, Pilar Nos, Javier Muñiz

**Affiliations:** ^1^ Gastroenterology Department, University Hospital, Ferrol, Spain.; ^2^ Gastroenterology Department, Puerta de Hierro University Hospital, Madrid, Spain.; ^3^ Gastroenterology Department, Bellvitge University Hospital; IDIBELL Barcelona University, Barcelona, Spain.; ^4^ Gastroenterology Department, Central University Hospital, Oviedo, Spain.; ^5^ Gastroenterology Department, San Pedro de Alacantara University Hospital, Cáceres, Spain.; ^6^ Gastroenterology Department, Poniente University Hospital, Almeria, Spain.; ^7^ Gastroenterology Department, Dr. Negrín University Hospital, Las Palmas de Gran Canaria, Spain.; ^8^ Gastroenterology Department, Marqués de Valdecilla University Hospital, Santander, Spain.; ^9^ Gastroenterology Department, La Fe University Hospital, Valencia, Spain.; ^10^ Gastroenterology Department, Miguel Servet University Hospital, Zaragoza, Spain.; ^11^ Gastroenterology Department, Gregorio Marañón University Hospital, Madrid, Spain.; ^12^ Gastroenterology Department, Instituto de Investigación Sanitaria Princesa and CIBERehd, La Princesa University Hospital, Madrid, Spain.; ^13^ Gastroenterology Department, Ramón y Cajal University Hospital, Madrid, Spain.; ^14^ Gastroenterology Department, Fuenlabrada University Hospital, Madrid, Spain.; ^15^ Instituto Universitario de Ciencias de La Salud e INIBIC, La Coruña University, La Coruña, Spain.

**Keywords:** behavioral health, e-health, home health monitoring, telehealth, telemedicine

## Abstract

***Objectives:*** Mobile apps are useful tools in e-health and self-management strategies in disease monitoring. We evaluated the Harvey–Bradshaw index (HBI) mobile app self-administered by the patient to see if its results agreed with HBI in-clinic assessed by a physician.

***Methods:*** Patients were enrolled in a 4-month prospective study with clinical assessments at months 1 and 4. Patients completed mobile app HBI and within 48 h, HBI was performed by a physician (gold standard). HBI scores characterized Crohn's disease (CD) as remission <5 or active ≥5. We determined agreement per item and total HBI score and intraclass correlation coefficients (ICCs). Bland–Altman plot was performed. HBI changes in disease activity from month 1 to month 4 were determined.

***Results:*** A total of 219 patients were enrolled. All scheduled assessments (385 pairs of the HBI questionnaire) showed a high percentage of agreement for remission/activity (92.4%, κ = 0.796), positive predictive value (PPV) for remission of 98.2%, and negative predictive value of 76.7%. High agreement was also found at month 1 (93.15%, κ = 0.82) and month 4 (91.5%, κ = 0.75). Bland–Altman plot was more uniform when the HBI mean values were <5 (remission). ICC values were 0.82, 0.897, and 0.879 in all scheduled assessments, 1 and 4 months, respectively.

***Conclusions:*** We found a high percentage of agreement between patients' self-administered mobile app HBI and in-clinic physician assessment to detect CD activity with a remarkably high PPV for remission. The mobile app HBI might allow a strict control of inflammation by remote monitoring and flexible follow-up of CD patients. Reduction of sanitary costs could be possible.

## Introduction

Crohn's disease (CD) is a chronic inflammatory disorder of the gastrointestinal tract of increasing incidence and prevalence, which requires life-long medical treatment to maintain remission and reduce digestive damage.^1,2^

The general course of CD is extremely unpredictable, characterized by periods of remission and activity. Clinical assessment of disease activity is important for early control of inflammation, to prevent disease progression and to improve long-term outcomes. Exacerbation is associated with symptoms, such as diarrhea, abdominal pain, and/or weight loss, but flare-ups rarely coincide with the outpatient clinic visits.^1,3^

The ideal approach for the control of the disease would be real-time monitoring of patients' symptoms. Telemedicine systems, based on patient-reported CD activity, could improve flare detection, help implement tight control strategies, and avoid unnecessary clinical evaluation of patients in remission, thus optimizing the use of the health care resources. To achieve these goals, a patient-friendly assessment tool, available for recording data in real-time would be required. Mobile applications represent a promising telemedicine tool to facilitate self-management in a new model of health care, where patients have a closer interaction with the physician team and are involved in their decision-making process.^4,5^

The importance of patient-reported measures in outcome evaluation and symptom management is increasingly recognized.^6,7^ Furthermore, the use of patient-reported outcome measures (PROMs) for evaluating effectiveness of inflammatory bowel disease (IBD) interventions is progressively supported by the U.S. Food and Drug Administration.^8,9^

The use of PROMs is promising in m-health apps, which are becoming the dominant method of e-health. Many studies have been undertaken to assess the use of the web and mobile applications for chronic disease management, such as hypertension, diabetes, chronic heart failure, and asthma.^10–13^ However, accurate e-monitoring tools for disease activity in IBD are scarcely developed.

Recently, the diagnostic performance of the Walmsley index self-administered by the patient has been evaluated through a Web-based platform to detect activity/remission in patients with ulcerative colitis (UC). A good diagnostic agreement has been obtained when compared with the in-clinic index utilization by the physician (CRONICA study).^14,15^ The advantage of the use of a standardized index in the monitoring of a disease is its established relationship with activity/remission. In CD, the most used indexes for in-clinic evaluation are the Crohn's disease activity index (CDAI)^16^ and the Harvey–Bradshaw index (HBI).^17^ Although CDAI is considered the gold standard index, it is not practical and is essentially limited to clinical trials. The HBI has a very good correlation with the CDAI with the advantage of being easy to use in clinical practice.^17–19^

Reports of diagnostic indexes to evaluate activity by the patients in CD using m-health apps are scarce, and, to the best of our knowledge, there are no studies comparing the diagnostic performance of a self-administration mobile app and an in-clinic standardized index such as the HBI.^20^

In the MediCrohn study, we aimed to evaluate if the HBI adapted to a mobile app, used as self-control questionnaire, is as useful as the original HBI questionnaire assessed by the physician to discriminate between activity and remission of CD. The HBI mobile app (HBImApp) could be used as part of the PROMs instruments in strategies of self-control and telemedicine.

## Methods

### Participants and Setting

Patients with established CD attending IBD outpatient clinics from April 2016 to June 2017 at 14 hospitals in Spain were invited to participate in a prospective, non-interventional, 4-month follow-up study, to assess the diagnostic performance (remission/activity of CD) of the self-administered HBImApp compared with the same index evaluated in-clinic by the gastroenterologist. The study was approved by the corresponding Clinical Research Ethics Committees.

The inclusion criteria were: (1) 18 years of age or older, (2) diagnosis of CD for >6 months confirmed by Lennard-Jones criteria, (3) familiarity with mobile apps or internet use, (4) a mobile phone with internet connection, and (5) signed informed consent for the study. Exclusion criteria were: (1) severe CD flares, (2) mental disorder or limitations that prevent accurate interpretation of the questionnaires, and (3) other relevant concomitant clinical conditions.

After inclusion in the study, patients and physicians received an explanation of the procedures and were trained by using a demonstration of the mobile application. To ensure privacy and security, patients had access to a personalized and private password-protected website (IBD training platform: www.educainflamatoria.com/entrenaeii), where the HBImApp version was available for completion. This platform was developed by IBD Unit of the Hospital-University Complex of Ferrol and CATCRONIC HEALTH Company.

Sample size calculation was based on a desired precision of ±4%, with a confidence level of 95%, for the global agreement between both measurements (patient and physician) of at least 80%. If each subject was evaluated at 1 and 4 months, 384 pairs of patient–physician questionnaires were needed and 192 patients must be enrolled. Assuming an expected 20% drop out rate, the number of subjects to be recruited was 230.

### Demographic Data and CD Characteristics

Baseline demographic data, the CD characteristics (disease location and behavior) and surgical history related to CD were recorded.

### HBI Mobile App

**Fig. 1.**
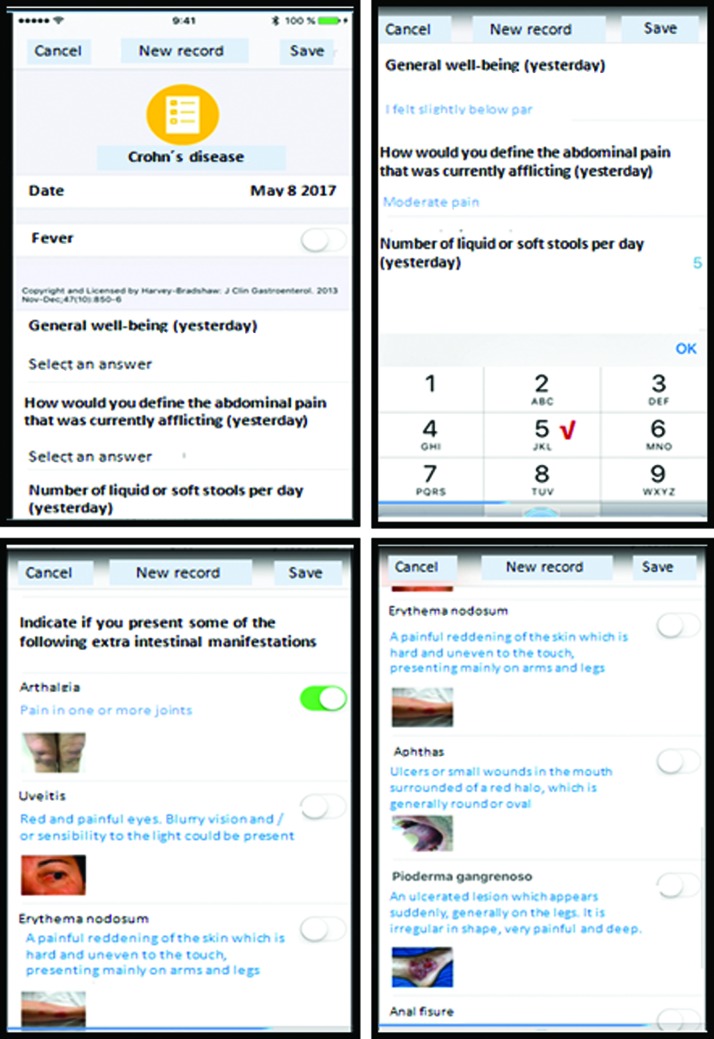
Screenshots of the HBI mobile app self-administered by the patients. Pictures of extraintestinal manifestations with a clear description of each of the hallmark symptoms are shown. Color images are available online.

The HBImApp is composed of 12 items in 5 domains: (1) general well-being, (2) abdominal pain, (3) number of liquid stools per day, (4) abdominal mass, and (5) extra intestinal manifestations of CD (arthralgia, uveitis, erythema nodosum, aphthous ulcer, pyoderma gangrenosum, anal fissure, new fistula, and abscess). Score ranges from 0 to 16 or more and the highest score depends on the number of liquid stools per day. To help the patients to identify the presence of extraintestinal manifestations, pictures were provided in the application, with a clear description of each of the hallmark symptoms (*Fig. 1*). Evaluation of comprehensiveness, clarity, and readability of app questionnaire was assessed in a group of 12 volunteers through cognitive interviews analysis. Patients found the test easy to understand, answered without supervision, and appeared to be comfortable with the images and questions. Aspects of translation equivalence were proved.

### Assessment of Patient-Reported HBI and in-Clinic HBI

The clinical assessment of the patients, including both mobile app self-evaluation and in-clinic evaluation by physicians, was performed at months 1 and 4. Short message service alerts were programmed as reminders to complete the HBI questionnaire through the app. Within 48 h, the patients attended an onsite hospital appointment where the HBI was performed by the gastroenterologist.

### Statistical Analysis

To evaluate the self-administered HBImApp, the HBI assessments by the physician at the outpatient clinic was considered the gold standard.

As primary objective of the study, we determined the percentage of total agreement between both tests to detect activity or remission of CD. HBI scores were treated as a dichotomous variable, scores <5 = remission and ≥5 = active disease.^18,19^ Cohen's κ coefficients were calculated to correct for the agreement expected by chance, with the following interpretation: poor (<0), slight (0–0.20), fair (0.21–0.40), moderate (0.41–0.60), substantial (0.61–0.80), and almost perfect (0.81–0.99).^21^ Negative predictive value (NPV), positive predictive value (PPV), and sensitivity and specificity to detect activity with 95% confidence intervals (CIs) were also calculated. Additionally, we examined agreement between scores of the HBI patient/physician on the total sum score and per item, percentages, and Cohen's κ were provided.

The level of agreement between the HBImApp assessed by the patient and the HBI assessed by the gastroenterologist was evaluated by intraclass correlation coefficients (ICCs), ranging from 0 to 1. An ICC of 0.75 and above is considered “excellent.” The difference between each pair of measurement was analyzed graphically as opposed to its mean by Bland–Altman plot methodology.

As a secondary objective, we evaluated the correlation between the changes in HBImApp from month 1 to month 4 assessed by the patient with the changes in the in-clinic HBI assessed by the physician. Changes in HBI questionnaire scores were categorized as follows: worsening (increase ≥3 points), stable (variations not exceeding 2 points), and improving (decrease ≥3 points), and the percentage of agreement and the Cohen's κ were calculated.^18,19^

## Results

Between April 2016 and June 2017, a total of 219 patients (116 females and 103 males), with mean age 36 ± 8 years were enrolled. Baseline characteristics are summarized in *Table 1*.

**Table 1.** Characteristics of the 219 Patients Included in the MediCrohn Study

Median age, years (IQR 25–75)	36 (32–41)
Male, *n* (%)	103 (47.03)
Smoking, *n* (%)	47 (21.66)
Educational level, *n* (%)	
Primary or secondary school	47 (21.46)
Professional studies	58 (26.48)
University degree	90 (41.10)
Internet use, *n* (%)	
Three times per week	209 (95.43)
Occasionally	10 (4.57)
Clinical characteristics	
Median age at diagnosis in years (IQR 25–75)	25 (22–28)
CD location, *n* (%)	
Ileal	94 (42.9)
Colonic	35 (15.98)
Ileocolonic	90 (41.1)
CD behavior, *n* (%)	
Inflammatory	143 (65.3)
Stricturing	65 (29.7)
Penetrating	11 (5.0)
Perianal CD present	38 (17.36)
Surgical history, *n* (%)	64 (29.22)
EIM, *n* (%)	60 (27.4)
Active disease (HBI ≥5) (%)	28.8
Medications at baseline, *n* (%)	
Biological treatment	72 (35.31)
Thiopurines	94 (46.09)
Steroids	12 (5.88)

CD, Crohn's disease; EIM, extraintestinal manifestation; HBI, Harvey–Bradshaw index; IQR, interquartile range.

A total of 385 pairs of questionnaires were valid to estimate the percentage of total agreement between the self-administered HBImApp and in-clinic gastroenterologist evaluation to detect activity or remission of CD. A total of 219 pairs of questionnaires from month 1 and 166 from month 4 were analyzed. Most of the patients filled out both mobile app HBI questionnaires, but 53 patients did not attend their medical check-up on the scheduled date at month 4 and were excluded from the second analysis. Results for the patients' and physicians' assessment of CD activity or remission in overall scheduled evaluations (A) and at 1- and 4-month evaluations (B and C, respectively) are shown in *Table 2*.

**Table 2.** Results of Patient's Self-Assessment Through a Mobile App HBI and Physician In-Clinic HBI Assessment

PATIENT SELF-ASSESSMENT	PHYSICIAN IN-CLINIC ASSESSMENT		TOTAL
	REMISSION (HBI <5)	ACTIVITY (HBI ≥5)	
A			
Remission (HBI <5)	277	5	282
Activity (HBI ≥5)	24	79	103
Total	301	84	385
B			
Remission (HBI <5)	154	2	156
Activity (HBI ≥5)	13	50	63
Total	167	52	219
C			
Remission (HBI <5)	123	3	126
Activity (HBI ≥5)	11	29	40
Total	*134*	*32*	*166*

A. All pairs of questionnaires (*n* = 385, κ = 0.796).

B. Pairs of questionnaires from month 3 (*n* = 219, κ = 0.824).

C. Pairs of questionnaires from month 6 (*n* = 166, κ = 0.753).

Percentage of agreement and predictive values between the self-administered HBImApp and in-clinic gastroenterologist evaluation with regard to the status of CD is shown in *Table 3*. The overall schedule evaluation percentage of agreement was 92.46% (95% CI 88.4–94.8) with a Cohen's κ coefficient of 0.796 (substantial agreement). Sensibility, specificity, PPV predicting clinical remission, and NPV are shown in *Table 3*.

**Table 3.** Percentage of Agreement and Predictive Values of Self-Administered Patient Mobile App HBI With Regard to the Gastroenterologist In-Clinic HBI Assessment

	ALL SCHEDULED EVALUATION (n = 385)	MONTH 1 EVALUATION (n = 219)	MONTH 4 EVALUATION (n = 166)
Overall percentage of agreement	92.46 (88.4–94.8)	93.15 (91.2–94.3)	91.5 (87.8–93.1)
Cohen's κ coefficient	0.796	0.824	0.753
Negative predictive value	76.7 (67.3–84.5)	79.4 (67.3–88.5)	72.5 (56.1–85.4)
Positive predictive value	98.2 (95.9–99.4)	98.7 (95.4–99.8)	97.6 (93.2–99.5)
Specificity	94 (86.7–98)	96.2 (86.8–99.5)	90.6 (75–98)
Sensibility	92 (88.4–94.8)	92.2 (87.1–95.8)	91.8 (85.8–95.8)

With the exceptions of Cohen's κ coefficient all values are presented as percentage (95% confidence interval).

The overall agreement from month 1 assessment was 93.15% (95% CI 91.2–94.3) with almost perfect agreement of Cohen's κ coefficient of 0.82. The study showed strong test–retest reliability with 91.5% (95% CI 87.8–93.1) of agreement at the 4-month assessment, Cohen's κ = 0.75 (substantial agreement) (*Tables 2 and 3*). There was good agreement for active versus inactive categorization at the two measurement time points. No differences by gender, age, internet use, educational level, or marital status were observed.

The mean of total HBI score of the 385 self-administered questionnaires was 3.3 (95% CI 2.9–3.8), and the mean value of those administered by the physician was 2.7 (95% CI 2.4–3.0) (*p* < 0.001). A large patient/physician agreement was observed, agreeing exactly on the questionnaire score in 56.4% of the cases. Differences of only 1 or 2 points were observed in 22.3% and 11.9% of cases, respectively. A difference of >2 points between the patient and the gastroenterologist HBI scores was observed in 9.4%.

**Fig. 2.**
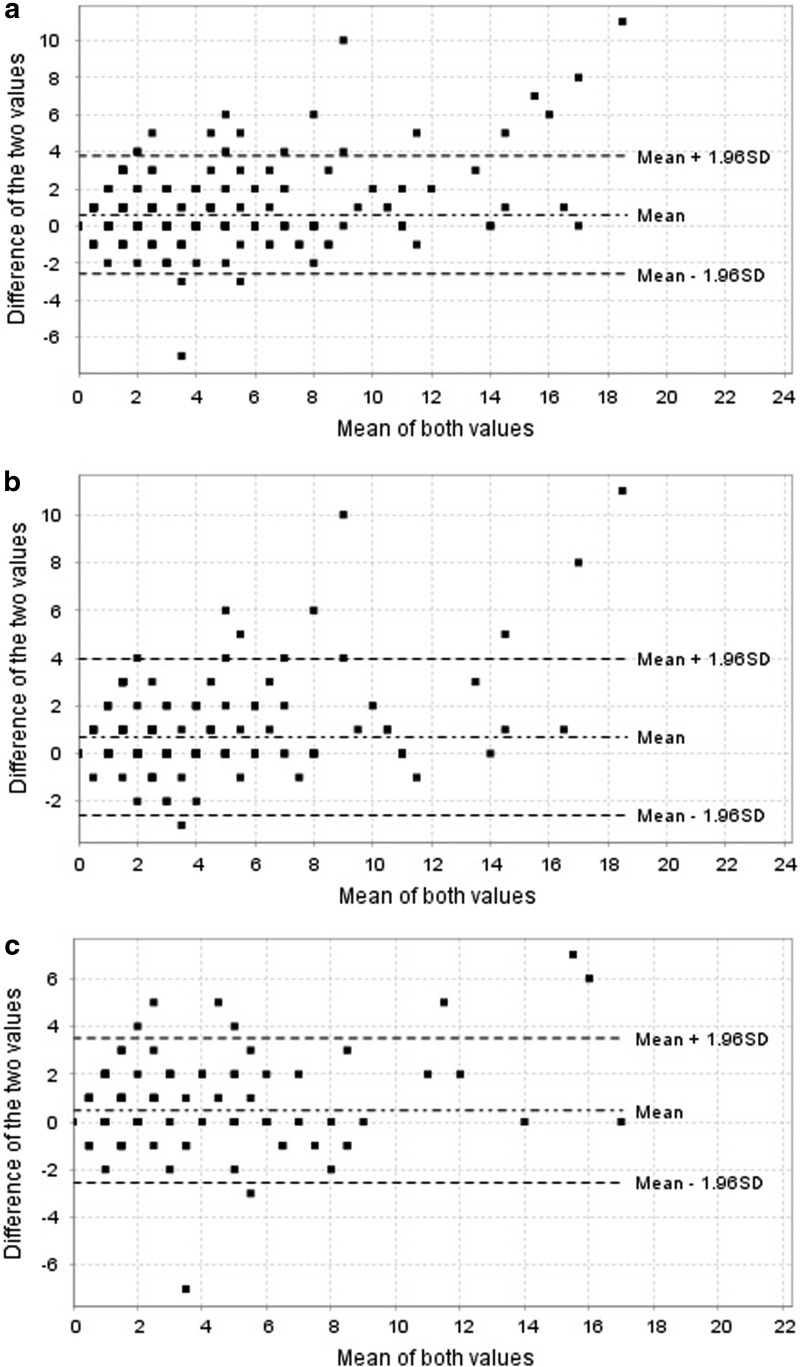
Bland–Altman plot for all schedule evaluation **(a)**, 1 month **(b)** and 4 month **(c)** of the agreement between the patient self-assessment HBI (app mobile) and in-clinic physician assessment. HBI, Harvey–Bradshaw index.

*Figure 2* shows the Bland–Altman plot of the difference between the assessment HBI (patient–physician) and the average of each pair of observations. The median of the difference between the score shows high dispersion in all scheduled, 1- and 4-month evaluations. The dots distribution is more uniform when the mean values are <5, which corresponds with the score for remission of the HBI.

Lastly, there is a high ICC globally, ICC (95% CI) = 0.82 (0.860–0.904), and both at month 1 = 0.879 (0.849–0.909) and at month 4 = 0.885 (0.853–0.918).

*Table 4* shows the percentage of agreement between patient/physician score in the different HBI domains. The lowest agreement was seen for number of liquid or soft stools per day domain (64.9% and moderate Cohen's κ value). Number of depositions per day was higher in patient score than in the physician 1.

**Table 4.** Percentage of Agreement in the Different Harvey–Bradshaw Index Domains

	OVERALL PERCENTAGE OF AGREEMENT	κ
1. General well being	89.6 (85.8–92.6)	0.742
2. Abdominal pain	87.7 (85.4–90.3)	0.706
3. Number of liquid or soft stools per day	69.3 (64.7–73.4)	0.567
4. Abdominal mass	91.6 (89.2–93.5)	0.491
5. Extraintestinal complications		
Arthralgia	90.1 (86.3–93.1)	0.782
Uveitis	95.3 (92.1–97.7)	0.529
Erythema nodosum	97.7 (94.5–98.2)	0.391
Aphthous ulcer	95.8 (92.8–97.6)	0.639
Pyoderma gangrenosum	100	1
Anal fissure	92.5 (89.1,94.7)	0.592
New fistula	96.9 (91.2–98.6)	0.318
Abscess	97.7 (94.5–99.1)	0.598

Data of agreement are presented as a percentage (95% confidence interval) and Cohen's κ values.

Extraintestinal manifestations were observed in 27.4% of the cases with a high percentage of agreement, all of them over 90%.

The ability to detect changes in disease activity (responsiveness) of app-administered HBI at 1 and 4 months was tested in a subset of 166 patients (*Table 5*). The percentage of agreement was 80.1% with a moderate correlation between the two questionnaires (Cohen's κ coefficient: 0.506).

**Table 5.** Agreement in the Change in Disease Activity from Month 1 to Month 4 Between the Patient Self-Assessed Harvey–Bradshaw Index Mobile App and the Gastroenterologist In-Clinic Assessment

CHANGE IN THE PHYSICIAN HBI	CHANGE IN THE PATIENT HBI			
	WORSENING	STABILITY	IMPROVING	TOTAL
Worsening, *n* (%)	7 (4.2)	7 (4.2)	1 (0.6)	15 (9)
Stability, *n* (%)	7 (4.2)	109 (65.7)	15 (9.0)	131 (78.9)
Improving, *n* (%)	0 (0.0)	3 (1.8)	17 (10.2)	20 (12)
Total, *n* (%)	14 (8.4)	119 (71.7)	33 (19.9)	166 (100)

Worsening: increased in HBI ≥3 points, Stability: HBI score variation not exceeding 2 points, Improving: decreased in HBI score ≥3 points (Vermeire Clinical Gastroenterology 2010).

## Discussion

e-Health technologies such as web-based interventions, virtual clinics, smartphone applications, and telemedicine are increasingly used for IBD patients' follow-up and continue to impact on health care. The link between e-health technologies with conventional clinical indexes and patient-reported outcomes could be cost effective and could facilitate the self-management of patients with IBD in a new model of patient-centered care.^20,22^

The use of PROMs to support routine IBD care is not widespread and suggests that existing questionnaires lack relevance to day-to-day decisions or are too cumbersome to administer.^23^ Recently, de Jong et al.^20^ performed a systematic review to identify available PROMs on IDB activity and whether they can be used effectively in routine practice, clinical trials, telemedicine systems, or value-based health care programs.

Development and validation of a new PROM may take several years; hence to adapt PROMs from existing indexes to e-health instruments, could be useful until more reliable instruments are available.^24^

We adapted the HBI to a mobile app because the data collection and calculation of HBI is simple, easily translatable into a patient-based questionnaire,^17,19,25^ and offers the possibility of capturing PROMs with minimal user burden.

In this study, we found that the patient-self-administered HBImApp questionnaire had a high agreement rate compared with the in-clinic physician-administered original HBI questionnaire to assess CD activity with a high accuracy (sensibility 92%; specificity 94%) and a noteworthy PPV for remission, suggesting that patients whose score with the HBImApp indicated remission will be very unlikely to have active disease. The reliability of the mobile app patient's-self-administered HBI was confirmed by the findings at the 3-month follow-up. The remarkably high PPV suggests that stable patients or those in remission might benefit from more flexible monitoring, including remote self-evaluations. Tools like HBImApp could allow better patient/hospital or patient/physician communication, potentially generating cost savings in the management of CD patients, and would be useful in routine medical care.^26^

While the evidence supporting the utility of telemedicine and internet-based interventions in IBD is emerging, the evidence supporting the efficacy of mobile phone apps in the CD setting is scarce.^20,22^ Although it was not a mobile app, Kim et al.,^27^ found that web-based diaries using the HBI can be useful in the monitoring of clinical disease activity in patients with CD, with good correlation between HBI completed through the web by the patient and the CDAI calculated by the medical staff. Recently, Van Deen et al.^9^ developed and validated a scoring system to monitor disease activity in patients with CD and UC that can be used with mobile technologies. Bennebroek et al.^25^ assessed the performance of the HBI filled out by the patient on paper compared with that of the treating physician, but to the best of our knowledge, this is the first time that diagnostic performance of HBImApp has been evaluated and reliability and responsiveness of the app was tested. Our results show higher percentage of agreement and Cohen's κ score regarding active disease versus remission with HBImApp than reported previously with paper questionnaires. Similar findings are reported by Larsen et al.^28^ using HBI touch screen, maybe related to a lower probability of unanswered questions on digital versions.

The ICC in all scheduled, 1- and 4-month pairs of questionnaires showed almost perfect correlations. Bland–Altman plot showed more concordance between the observers in the low score of the scale suggesting better agreement when the patient is in remission.

The high percentage of patient/physician agreement on item-level HBI scores was found. The domains well-being and abdominal pain showed the highest percentage of agreement and the least agreement is shown in the number of stools per day. Number of depositions per day is higher in patient score than in that of the physician. Many patients referred to total depositions (not only liquid or loose stools), but probably this item will improve with the use of the mobile app HBI by the patient. High agreement was observed in the domain abdominal mass, in spite of the fact that it was evaluated by the patient without physician intervention. Bennebroek et al.^25^ developed a modified patient HBI, omitting the physical examination of abdominal mass, assuming that patients cannot adequately examine themselves. They found high agreement to characterize CD activity between the modified HBI and the original HBI in-clinic assessed by the physician, suggesting that this item could be omitted if the HBI will be used as a PROM instrument.

To achieve our secondary objective, we scheduled a prospective follow-up, with 2 evaluations of the HBI 3 months apart. We found a high patient/physician agreement in the changes in disease activity from month 1 to month 4. These findings confirmed the usefulness of the HBImApp as a user-friendly tool of e-health that allows the remote self-monitoring of disease activity. Self-management strategies through e-health tools provide the patient with opportunities for easy access to medical care and individualized treatment in a medical system with increasing patient-centered care focus. An e-Health-Enhanced Chronic Care Model is being proposed to improve health care quality. e-Health tools can be used to increase efficiency when patients manage their own chronic illnesses.^29^

One of the strengths of this study is that the patients were familiar with HBI questions, therefore, differences in answers between paper questionnaires and mobile app were not confounded by difficulties in understanding the questions. With the HBImApp it is not possible to proceed without answering, which probably decreased the number of missing data in the questionnaire. The mobile app was completed within the 48 h before physician evaluation, thereby minimizing memory biases.

The current study has several limitations. First, a possible selection bias because the patients enrolled must be familiar with mobile apps or internet use and have a mobile phone with internet connection. Second, we failed to collect 53 pairs of HBI questionnaire in the second assessment because patients did not attend their medical check-up on the scheduled date. Third, the study may not be representative for elderly patients, because we did not include enough patients above 65 years to investigate app use in this group of age. Finally, the mobile app was validated against the original HBI instead of endoscopy, gold standard to validate PROM measuring disease activity, because HBI do not accurately reflect endoscopic disease activity in patients with CD.

## Conclusions

Our results showed a high percentage of agreement between self-administered HBImApp and in-clinic physician assessment to categorize activity/remission in CD with a remarkably high accuracy and high PPV for remission. Moreover, the HBImApp showed good reliability and responsiveness to changes in disease activity. In this regard, it could be considered as an adequate m-Health PROM instrument for CD activity monitoring for use in clinical practice. Possible beneficial effects in patient disease control, less frequent outpatient's visits of patients in remission, and reduction of sanitary costs must be considered.
